# Electrophysiological and biophysical perspectives on the clitoral corpus cavernosum and its role in female sexual arousal disorder

**DOI:** 10.3389/fphys.2025.1626675

**Published:** 2025-09-01

**Authors:** Chitaranjan Mahapatra, Jagatpati Raiguru, Kasetty Lakshminarasimha, Ahmed Al-Emam, Maher Ali Rusho

**Affiliations:** ^1^ Paris Saclay Institute of Neuroscience, Paris Saclay University, Saclay, France; ^2^ Department of Electronics and Communication Engineering, Aditya University, Surampalem, India; ^3^ Department of Electronics and Communication Engineering, SVR Engineering College, Nandyal, India; ^4^ Department of Pathology, College of Medicine, King Khalid University, Asir, Saudi Arabia; ^5^ Department of Biomedical Engineering, University of Colorado, Boulder, CO, United States

**Keywords:** clitoris, smooth muscle, biophysics, ion channel, calcium dynamics, membranepotential, electrophysiological model

## Abstract

The clitoris is a vital part of the female sexual system, playing a crucial role in sexual satisfaction and overall sexual health. During arousal, the smooth muscle within the corpus cavernosum of the clitoris relaxes, which increases blood flow and causes the clitoral tissues to swell and firm. This vascular engorgement is not only essential for clitoral erection but also enhances the pleasurable sensations during sexual activity. However, conditions such as pelvic floor disorders, Peyronie’s disease, neuromuscular disorders, and hormonal imbalances can lead to dysfunction in the smooth muscle of the corpus cavernosum, significantly impacting female sexual function. The contractile behavior of these smooth muscles is governed by intricate cellular and subcellular processes, particularly the generation of intracellular electrical activities, with calcium influx playing a central role. This calcium influx is mediated through voltage-dependent calcium channels and calcium release from intracellular stores. Despite the critical importance of these mechanisms, comprehensive studies on the biophysical aspects of smooth muscle electrophysiology are limited, likely due to their complexity. This review seeks to investigate the cellular electrophysiological mechanisms underlying the electrical excitability of corpus cavernosum smooth muscle and to understand the biophysical aspects of clitoral muscle contraction disorders. It also proposes a first conceptual model to guide future research, with the aim of supporting the development of more effective treatments and enhancing female sexual health.

## 1 Introduction

**FIGURE 1:**
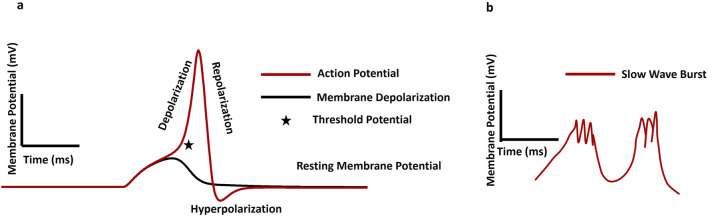
**(a)** Schematic diagram of a single isolated smooth muscle in relaxed and contracted states. **(b)** Illustrates the internal mechanisms during these states, with the symbol P indicating phosphorylation.

The recognition of women’s sexual pleasure as a fundamental human right is crucial for overall wellbeing and gender equality. This right includes the ability to control one’s sexuality, access to sexual and reproductive health services, and the enjoyment of sexual pleasure without discrimination or violence. International human rights frameworks, such as those established by the United Nations, highlight the significance of sexual rights and their connection to autonomy, privacy, and health (Starrs and Anderson, 2016). When sexual health and pleasure are neglected, it can lead to broader societal issues, including reduced productivity, higher healthcare costs, and strained interpersonal relationships. Addressing sexual health disparities can alleviate some of these economic burdens by improving mental health, reducing the incidence of sexually transmitted infections, and enhancing overall life satisfaction (World Health Organization, 2010). Promoting fundamental research on sexual wellbeing is a matter of personal health and a means of fostering a more equitable and economically robust society. The clitoris, often described as the epicenter of women’s sexual pleasure, is a highly sensitive organ located at the top of the vulva, beneath the clitoral hood (Pauls, 2015). With thousands of nerve endings, it is solely dedicated to pleasure, making it a key player in female arousal and orgasm (Mazloomdoost and Pauls, 2015). Though small in size, the clitoris is a uniquely complex organ capable of significant engorgement and intense sensation during arousal, underscoring its central role in women’s sexual pleasure and the need to better understand and prioritize it in sexual health. The clitoral cavernous smooth muscles (CSM) around the clitoris play an essential role in sexual arousal and orgasm (Mazloomdoost and Pauls, 2015). The CSM, integral to the corpus cavernosum, relaxes during sexual stimulation to permit blood engorgement, causing the spongy tissue to swell and become erect. Conversely, the contraction of the CSM facilitates the return of blood flow out of the erectile tissue, leading to detumescence, or the return of the clitoris to its flaccid state after sexual activity. Smooth muscle, an involuntary and non-striated tissue found throughout the body, is classified into single-unit and multi-unit types, with contraction patterns further divided into tonic and phasic forms (Banerjee, et al., 2023; Reho et al., 2014). Tonic smooth muscles, such as those in blood vessel walls, maintain slow, sustained contractions over long periods, while phasic smooth muscles, found in the gastrointestinal and urogenital tracts, contract rapidly and rhythmically to support functions like peristalsis (Patel and Satish, 2006; Makhlouf and Murthy, 2015). Excitation-contraction coupling (E-C coupling) serves as a vital link between the electrical excitation of the muscle cell membrane (sarcolemma) and the initiation of muscle contraction (Rall, 2024). Calcium ions (Ca^2+^) play a crucial role in initiating smooth muscle contraction by activating key proteins in the contractile pathway. The process begins when an action potential (AP) on the sarcolemma causes an influx of Ca^2+^ into the cytoplasm. These ions bind to calmodulin, inducing a conformational change that activates Myosin Light Chain Kinase (MLCK). Activated MLCK then phosphorylates the myosin regulatory light chain (RLC), enabling myosin heads to bind to actin filaments. The interaction between myosin and actin causes the filaments to slide past each other, triggering a series of conformational changes that result in muscle contraction (Wang and Raunser, 2023). Concurrently, Myosin Light Chain Phosphatase (MLCP) dephosphorylates RLC, balancing contraction with relaxation. Figure 1 illustrates these cellular and molecular events: panel (a) shows how an AP raises intracellular Ca^2+^ levels, switching the smooth muscle cell from a relaxed to a contracted state; panel (b) details the molecular pathway where Ca^2+^-calmodulin activates MLCK, leading to myosin phosphorylation and contraction. Relaxation occurs as MLCP removes phosphate groups from myosin, disrupting its interaction with F-actin. The symbol “P” in Figure 1b denotes phosphorylation.

The phasic contractile characteristics of CSM cells are controlled by intracellular Ca^2+^, facilitated through voltage-dependent Ca^2+^ channels (VDCC) and Ca^2+^ release from intracellular stores. The intracellular Ca^2+^ concentration is also modulated by the coordinated actions of ion channels, neurotransmitters, and hormonal signals, leading to cyclic changes in CSM tone and elasticity. During sexual arousal, stimulation of the dorsal nerves induces relaxation of the clitoral CSM, facilitating intercourse (Munarriz et al., 2003). Disorders related to CSM arise from irregular contractions or changes in their mechanical properties, which can significantly impact sexual health and function. For example, aging causes changes in the clitoral cavernosal erectile tissue, which may play an important role in the pathophysiology of age-associated female sexual arousal disorders (Berman, 2005). Persistent ischemia in the clitoral cavernosal tissue leads to considerable fibrosis and a reduction in smooth muscle, potentially contributing to the development of female sexual arousal disorders (Berman, 2005; Park et al., 2001). Due to its vital role in female sexual health, a thorough understanding of clitoral smooth muscle contraction is crucial. Investigating the biophysical mechanisms behind this process enhances our grasp of muscle physiology and provides a foundation for developing targeted treatments for smooth muscle-related conditions, such as female sexual arousal disorders (FSAD). Despite the relevance of these mechanisms, research remains fragmented, and studies focusing specifically on the biophysics of clitoral CSM are notably limited. Although various experimental techniques—such as whole-cell and current-clamp recordings, immunohistochemistry, confocal imaging, and muscle tension analyses—have been extensively applied to other types of smooth muscle, clitoral CSM has not been thoroughly investigated using these approaches (Noguchi et al., 2021; Murayama et al., 2012). The interdisciplinary nature of biophysics, requiring both biological and physical science expertise, may contribute to the scarcity of integrative reviews in this area (Hille, 2022). Furthermore, existing literature only tends to address isolated aspects of smooth muscle physiology, lacking a unified perspective that connects electrophysiology, Ca^2+^ dynamics, and force production in the clitoral context. This review aims to fill that critical gap by offering a holistic synthesis of experimental evidence related to the biophysical properties of clitoral CSM contraction. Furthermore, a central objective of this work is to establish a conceptual model that captures the key cellular mechanisms driving clitoral smooth muscle electrophysiology function. Such a model is vital not only for advancing foundational science but also for bridging the gap between basic research and clinical application, ultimately contributing to more effective therapeutic strategies for female sexual health.

## 2 Materials and methods

In pursuit of a comprehensive and in-depth review of clitoral smooth muscle electrophysiology, we conducted an unrestricted MEDLINE search via PubMed, including all English-language studies regardless of publication date (Katchamart et al., 2011; Sampson et al., 2016). Articles written in languages other than English and research that repeated data from other sources were not included. Original research articles that evaluated the impact of ion channels and calcium dynamics on the excitability and contraction of smooth muscles were the main focus of our inclusion criteria. These included experimental research, prospective observational studies, retrospective cohort studies, case-control studies, randomized and non-randomized clinical trials, and review articles. To guarantee a thorough grasp of the subject, every chosen article was carefully analyzed, and extra references were checked. Due to the limited availability of direct experimental data on CSM electrophysiology, we systematically extrapolated findings from studies on other smooth muscle types to draw informed inferences about CSM. This approach was carefully structured to maintain scientific rigor and ensure meaningful interpretation. A key step in this process was identifying both the physiological and functional parallels between CSM and other smooth muscles, such as gastrointestinal, vascular, and urinary tract smooth muscles. These tissues share fundamental characteristics, including similar ion channel activity, Ca^2+^ signaling pathways, modes of contraction, and patterns of intracellular electrical behavior (e.g., slow waves, action potentials, depolarization, and hyperpolarization). To elucidate the likely electrophysiological mechanisms in CSM, we conducted a comprehensive literature review of these related systems, drawing on both classical and contemporary sources published between 1971 and 2025. Although some of the references are historically significant and foundational, the review also integrates recent advances to provide an updated and well-rounded perspective. We begin with an overview of intracellular electrophysiology, laying the groundwork for how electrical signals are generated and propagated in CSM. This is followed by a detailed exploration of ion channel biophysics, gap junctions, and varicosities, highlighting the cellular and subcellular mechanisms that regulate excitation and coordination within the tissue. We also examine the role of interstitial cells of Cajal (ICCs), drawing parallels from other smooth muscle systems to propose their potential modulatory role in CSM. Next, we discuss calcium dynamics, a key driver of smooth muscle contraction, before introducing a membrane potential conceptual model specific to CSM to provide a theoretical framework for interpreting electrophysiological behavior. To support these insights, we review relevant experimental and computational techniques used to study smooth muscle contraction, emphasizing their applicability to CSM. The sections on intracellular electrophysiology, ion channel biophysics, gap junctions, varicosities, interstitial cells of Cajal, and calcium dynamics are first discussed in the context of smooth muscle in general, and then specifically applied to clitoral smooth muscle. Finally, the review concludes with pharmacological implications and future directions, identifying therapeutic targets and research gaps, followed by a conclusion that synthesizes the key findings and underscores their relevance to FSAD. This structured flow was designed to ensure both depth and coherence in addressing the electrophysiological and biophysical basis of CSM function.

## 3 Intracellular electrophysiology

**FIGURE 2:**
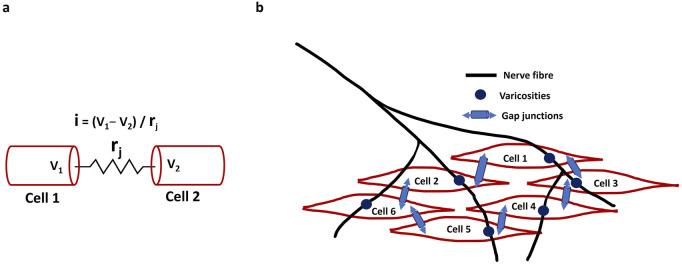
**(a)** Shows the slow wave pattern with bursting, whereas **(b)** shows the membrane depolarization (black solid line), AP (red solid line), depolarization, repolarization, threshold potential (star mark), and resting membrane potential.

Key electrical terms—membrane potential, depolarization, hyperpolarization, slow waves, and action potentials—are fundamental to excitable cell electrophysiology, reflecting ion gradients and membrane permeability that drive essential functions in neurons and muscle cells (Hille and Catterall, 2012). The membrane potential refers to the voltage difference across a cell’s plasma membrane, resulting from the distribution of ions between the inside and outside of the cell and the membrane’s selective permeability to these ions. This voltage difference is typically measured in millivolts (mV). The resting membrane potential (RMP) is the voltage difference across the membrane of a cell at rest, primarily established by the distribution of sodium (Na^+^), Ca^2+^, potassium (K^+^), chloride (Cl^−^), and other ions (Mahapatra et al., 2025). The Na^+^/K^+^-ATPase pump actively maintains ionic gradients essential for the RMP, working in conjunction with ion channels and ionic distributions (Huang et al., 2024). Voltage stability is also indirectly impacted by the capacitance and permeability of the cell membrane, which are determined by its lipid composition. Depolarization is a key process in cellular excitability, marked by a decrease in membrane potential that makes the cytosol less negative relative to the RMP. In contrast, hyperpolarization increases the membrane potential, making the intracellular environment more negative than the RMP. An AP is a rapid, temporary change in membrane potential that enables signal transmission via ion flow across the membrane. While single AP firing generates isolated spikes in response to stimuli, burst firing produces multiple APs in quick succession followed by resting period. (Leleo and Segev, 2021). Several studies have emphasized membrane potential’s role in regulating diverse cellular functions, including the cell cycle, cell volume, proliferation, and other sub-cellular activities (Yang and Brackenbury, 2013; Rosendo-Pineda et al., 2020; Becchetti 2011). Smooth muscle cells display distinctive electrical firing activity patterns, such as plateau potentials, slow waves (SW), pacemaker potentials, and action potentials (Mahapatra and Kumar, 2024). The plateau potentials (sustained depolarizations) are particularly prominent in certain types of smooth muscle, such as vascular smooth muscle found in blood vessels. By prolonging the duration of depolarization, plateau potentials enable sustained contraction, which is essential for functions like maintaining blood pressure and regulating blood flow (Ottolini and Sonkusare, 2021; Gonzales et al., 2010). SWs, also termed basic electrical rhythm, manifest as rhythmic fluctuations in smooth muscle cell membrane potential, generated by specialized pacemaker cells like the interstitial cells of Cajal (ICC) in the GI tract (Van et al., 2010). They serve as electrical pacemakers, setting the rhythm for contractions by initiating excitatory events that can trigger APs in neighboring smooth muscle cells. Conversely, pacemaker potentials are spontaneous depolarizations occurring regularly in pacemaker cells due to their intrinsic rhythmicity. In smooth muscle tissues such as the urinary bladder, pacemaker potentials regulate the timing and coordination of contractions essential for functions like urination. APs are generated in various smooth muscles—including the vas deferens, urethra, ureter, uterus, and bladder—and can be triggered by neurotransmitters, hormones, mechanical stretch, or shifts in extracellular ions (Mahapatra and Kumar, 2024). These APs propagate via gap junctions, enabling synchronized contractions, and are typically longer in duration due to slower kinetics of voltage-gated ion channels (Dixon et al., 2022). Interestingly, some smooth muscles also display spontaneous depolarization, hyperpolarization, and APs, contributing to their unique physiological roles (Ding et al., 2023). Latency of AP refers to the brief delay between the application of a stimulus and the initiation of the AP in the excitable cell (Title et al., 2025). The conduction velocity is also a critical cable property that determines the rate at which the membrane potential propagates along the cell membrane (Park, 2023). This velocity is influenced by factors like the cell’s diameter and the presence of myelin sheaths, which insulate the cell and facilitate faster signal transmission through saltatory conduction (Fry and Jabr, 2010). The conduction velocity is crucial because it determines the efficiency and speed of neural communication, affecting how quickly the nervous system can respond to stimuli, coordinate complex behaviors, and maintain vital functions like muscle contraction and reflex actions (Park, 2023). Figure 2a presents a simulated result showcasing various aspects of membrane activity: membrane depolarization (depicted by the black solid line), action potential (shown by the red solid line), phases of depolarization and repolarization, threshold potential (indicated by a star mark), and the resting membrane potential. Figure 2b illustrates the pattern of a slow wave accompanied by bursting activity.

The value of RMP and the kinds of electrical characteristics produced in the major smooth muscle cells are shown in Table 1.

**TABLE 1:** RMP values in different smooth muscle cells.

Tissue type (smooth muscle)	Resting membrane potential (mV)	Action potential (AP)/Slow wave (SW)	References
Uterine	−35 to −80	AP	Aguilar and Mitchell (2010)
Ureter	−30 to −70	AP	Bastiampillai et al. (2020)
Urinary bladder	−45 to −55	AP	Mahapatra and Kumar (2024)
Urethra	−37 to −45	AP	Kyle, B. D. (2014)
Vas deferens	−50 to −60	AP	Mahapatra et al. (2018a)
Seminal vesicles	−50 to −60	SW	Kajimoto et al. (1972)
Colon (GI tract)	−40 to −80	SW	Sanders, K. M. (2019)
Pulmonary artery Aorta	−60 to −85	SW	Santos-Gomes et al. (2022)
Aorta	−50	SW	Cauvin et al. (1984)
Portal vein	−50	SW	Kuriyama et al. (1971)
Gall bladder	−45	AP	Jaggar et al. (1998)

It has been demonstrated that electrical stimulation, through the release of various neurotransmitters from the parasympathetic innervation, can induce both contraction and relaxation in CSM strips, suggesting the presence of nerve-mediated pathways that regulate muscle activity (Angulo and Hannan, 2022). Contractions in CSM are believed to be driven by noradrenaline (NA) released from sympathetic nerves. The function of neurotransmitter release in smooth muscle regulation is also highlighted by the theory that cholinergic neurons mediate relaxation through the muscarinic acetylcholine receptor M3 (Hashitani et al., 2005). Additionally, nitric oxide (NO), produced by non-adrenergic, non-cholinergic (NANC) neurons, is another key neurotransmitter that promotes CSM relaxation (Craven et al., 2004). Consistent evoked and spontaneous electrical activities were recorded extracellularly in the CSM, although the specific characteristics of these activities were not quantified. The latency and amplitude of the evoked extracellular electrical activity from the clitoris muscle are reported as 220 µV and 1731 ms, respectively (Yilmaz et al., 2004). Shafik’s laboratory has recorded both SWs and APs bursts from CSM cells using electromyography techniques (Shafik et al., 2009). They have proposed that the RMP of CSM and vaginal smooth muscle may be comparable to that of uterine smooth muscle, approximately −50 mV (Shafik et al., 2009; Shafik et al., 2004). A study by the Hashitani group has revealed RMP of rabbit CSM lies within the range −50 mV to −41 mV (Hashitani et al., 2005). In another electromyographic study, the basal value of the CSM slow waves’ frequency, amplitude, and conduction velocity are recorded as 4.1 cycles/min, 0.42 mV, and 4.7 cm/s, respectively (Shafik et al., 2008). However, these values fluctuated throughout the micturition cycle, and the slow waves were often followed by or superimposed with bursts of rapid spike activity or action potentials.

## 4 Ion channel biophysics

Ion channels are broadly classified as voltage-gated, ligand-gated, and mechanically-gated ion channels, each exhibiting diverse structural and functional properties (Mahapatra et al., 2025). In excitable cells like neurons and muscle fibers, voltage-gated ion channels—such as Na^+^, K^+^, Cl^−^, and Ca^2+^ channels—respond to changes in membrane potential, enabling the rapid generation and propagation of APs (Alexander et al., 2017). While voltage-gated channels are primarily responsible for AP propagation, ligand-gated ion channels—activated by specific neurotransmitters or ligands, such as nicotinic acetylcholine receptors (Lemoine et al., 2012)—mediate synaptic transmission and postsynaptic responses, influencing cellular excitability and signaling pathways. Excitable cells also contain mechanically-gated ion channels, which open in response to physical stimuli such as pressure or stretch and transform mechanical signals into electrical signals (Douguet and Honoré, 2019). Meanwhile, through activating Ca^2+^-sensitive signaling pathways, intracellular Ca^2+^ levels can either directly or indirectly affect channel activity in smooth muscle cells (Ottolini and Sonkusare, 2021). Several studies have shown that Na^+^ ions often serve as secondary carriers of inward current in tissues where rapid activation is less critical, such as cardiac and smooth muscles (Mahapatra et al., 2025). These channels play key roles in regulating contractility, intracellular Ca^2+^ levels, and membrane potential. In smooth muscle tissues, L-type and T-type Ca^2+^ channels are essential for generating inward current during depolarization and action potential formation (Mahapatra and Rohit, 2017). In particular, by preventing cholinergic nerve stimulation and acetylcholine activity at muscarinic receptors, L-type Ca^2+^ channel antagonists, such nifedipine, efficiently prevent smooth muscle contraction. Voltage-gated K^+^ (Kv) channels are essential for repolarizing the membrane during an AP generation, thus helping to maintain the RMP and control cellular excitability (Nakajo and Go, 2024). Inward rectifier channels, another type of voltage-gated K^+^ channel, are prevalent in various smooth muscle cells. These channels, termed “rectifiers,” preferentially allow K^+^ ions to flow into the cell rather than out. Dependent on voltage after being triggered by intracellular Ca^2+^ and/or modifications in membrane potential, Ca^2+^-activated K^+^ (KCa) channels produce a hyperpolarizing after-potential that affects the firing rate of APs (Nam et al., 2023; Mahapatra and Manchanda, 2018b; Mahapatra et al., 2018). According to their conductance, these channels can be divided into three primary categories: small, medium, and large. With unit conductances ranging from 400 to 800 pS, the big conductance channels—also referred to Maxi K channels—are important modulators of neuronal excitability and smooth muscle contraction (Dudem et al., 2021). This activation increases potassium ion efflux, causing membrane hyperpolarization and promoting smooth muscle relaxation. Compared to their large counterparts, small and intermediate conductance channels are less well understood. Their unit conductances range from 2 to 20 pS for small conductance channels and 20–85 pS for intermediate conductance channels (Petkov 2012). These channels are solely controlled by internal Ca^2+^ ions and are more responsive to intracellular Ca^2+^ levels. Another type of K^+^ channel, ATP-sensitive K^+^ (K_ATP_) channels, where the increase in ATP inhibits the regulatory elements, causing the channels to close, and are essential for smooth muscle function (Mahapatra et al., 2016). By preventing the formation of APs, they allow for subtle adjustments in membrane potential, which is critical for various vascular responses (Singareddy et al., 2022). The repolarization of APs in smooth muscles also depends on voltage-sensitive K^+^ channels, also referred to as KDR channels (Tsuji-Tamura et al., 2021). These channels regulate the K^+^ ion outflow, which impacts the frequency and length of APs and, ultimately, the contraction and relaxation of muscles. Transient receptor potential (TRP) channels are non-selective cation channels regulated by phosphatidylinositol signaling, with subtypes like TRPC3, TRPC6, and TRPC7 predominantly expressed in smooth muscles as receptor-operated channels involved in pain perception and cell cycle control (Geng and Ma, 2025). TRPV1 is a Ca^2+^-permeable channel activated by heat and low pH, while TRPV2 responds to mechanical stretch, TRPV3 and TRPV4 to high temperatures, and TRPV4 additionally to hypotonic conditions causing cell swelling. TRPM1 is broadly expressed with unclear function; TRPM2 and TRPM3 are Ca^2+^-permeable, activated by ADP-ribose/NAD and hypotonicity, respectively, whereas TRPM4 and TRPM5 are monovalent-selective channels found mainly in the kidney, CNS, and smooth muscle (Ji and McCulloch, 2021). Early studies on striated muscle suggested that chloride ions (Cl^−^) distribute passively due to their equilibrium potential being close to the RMP (∼–65 mV) (Jentsch and Michael, 2018), indicating high Cl^−^ permeability that helps stabilize the RMP and regulate responses to Cl^−^ channel activity—crucial for maintaining normal physiological functions in smooth muscle cells (Wray et al., 2021). An interesting cellular mechanism that maintains Ca^2+^ homeostasis is the store-operated system. A considerable amount of Ca^2+^ is reduced from the cell when intracellular Ca^2+^ stores in the sarcoplasmic reticulum (SR) are activated and depleted (Pearce et al., 2023). The cell starts a Ca^2+^ influx from the extracellular environment to replenish these exhausted resources. Specialized channels called Ca^2+^ release-activated channels (CRAC) help to assist this influx. I_CRAC_, a particular subtype of these channels, has unique properties, including being highly selective for Ca^2+^, inwardly rectifying, and non-voltage sensitive (Emrich et al., 2022; Potier et al., 2009).

Numerous studies have shown that the corpus cavernosum contains a diverse array of ion channels—including voltage-gated K^+^ channels (Kir, Kdr, KATP, Kv1), voltage-gated Na^+^ channels, voltage- and Ca^2+^-gated Cl^−^ channels, and cyclic GMP–stimulated large-conductance KCa channels—along with CRAC, TRP, leak, and T- and L-type voltage-gated Ca^2+^ channels, all of which contribute to excitability, contraction, and signal transduction (Gragasin et al., 2004; Malysz et al., 2002; McCloskey et al., 2009; Ückert et al., 2017; Chen et al., 2004). In contrast, evidence from clitoral smooth muscle is currently limited to BKCa, SKCa3, and TRPA1 channels (Gragasin et al., 2004; Chen et al., 2004; Ückert et al., 2017).

In addition, hormonal status, particularly estrogen deficiency, plays a pivotal role in modulating the electrophysiological and contractile properties of CSM cells by altering membrane potential and ion channel expression. Estrogen enhances endothelial nitric oxide synthase (eNOS) activity, thereby increasing nitric oxide (NO) production, which promotes smooth muscle relaxation through activation of KCa channels and subsequent membrane hyperpolarization (Creighton et al., 2004). In contrast, estrogen deficiency downregulates KCa channel expression and upregulates L-type Ca^2+^ channels, leading to membrane depolarization, elevated intracellular Ca^2+^, and increased contractility of CSM cells (Gragasin et al., 2004). Progesterone may antagonize estrogenic effects by reducing KCa channel activity, while testosterone has been shown to potentiate both KCa and Kv channels, further influencing CSM tone and responsiveness (Moore et al., 2019). These hormonal modulations of ion channel dynamics contribute to vascular dysfunction in hypoestrogenic states, such as menopause, and are implicated in the pathophysiology of female sexual arousal disorders (Kaufman et al., 2023). While much of the current research on CSM physiology focuses on NO and cyclic GMP pathways, several subcellular signaling mechanisms remain underexplored and could significantly enrich future investigations. Notably, second messengers such as cyclic AMP (cAMP) and inositol trisphosphate (IP_3_), along with protein kinase cascades like PKA, PKC, and MAPK, are known to regulate smooth muscle tone and vascular responses in other genital tissues but have received limited attention in CSM studies. For instance, PKC activation via DAG and IP_3_-mediated Ca^2+^ release has been implicated in genital smooth muscle contraction and may play a role in clitoral vasocongestion and arousal physiology. Additionally, cyclic AMP-dependent pathways involving PDE4 and PKA could modulate CSM relaxation, as suggested by findings in adjacent urogenital tissues (Ückert, S et al., 2011). Investigating these cascades may uncover novel therapeutic targets for female sexual dysfunction and broaden our understanding of clitoral signal transduction beyond the NO/cGMP axis.

## 5 Gap junction, varicosities, and interstitial cells of Cajal

**FIGURE 3:**
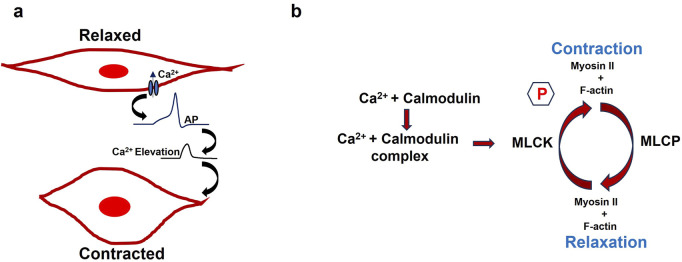
**(a)** Diagram showing how two cells are connected by a gap junction Cells 1 and 2. The gap junction resistance between two cells is represented by the r_j_ value, and the membrane potentials of cells 1 and 2 are denoted by V1 and V2. **(b)** Varicosities from nerve cells are distributed throughout the smooth muscle syncytium, where gap junctions connect the individual cells.

Gap junctions are specialized cellular structures that enable direct communication between adjacent cells by forming high-conductance channels for the passage of ions and small signaling molecules. Constructed from connexin proteins, these gap junctions enable the swift and synchronized transmission of electrical and chemical signals essential for smooth muscle coordination and function (Appukuttan et al., 2021). Connexins belong to a diverse multigene family comprising over 20 isoforms in humans, categorized into five subfamilies based on sequence similarity: alpha (GJA), beta (GJB), gamma (GJC), delta (GJD), and epsilon (GJE). Commonly expressed connexins in smooth muscle tissues include Cx43 (GJA1), Cx26 (GJB2), and Cx45 (GJC1), among others (Ke, et al., 2025; Beyer and Berthoud, 2018; Cardouat, et al., 2024). The electrically coupled network due to gap junction connections behaves similarly to a syncytium, playing a crucial role in various physiological processes. Gap junctions allow depolarizing currents to pass directly between adjacent smooth muscle cells, enabling the spread of electrical signals. If the transmitted depolarization reaches the threshold potential in a neighboring cell, it can trigger an action potential, promoting synchronized excitation across the tissue. Figure 3 is a schematic presentation illustrating the detailed biophysical mechanism of a gap junction. The gap junction resistance by r_j_ between two cells (Cell 1 and Cell 2) is depicted in Figure 3a. The intracellular membrane potentials of these two cells are V_1_ and V_2,_ respectively. The current ‘i’ flowing from higher membrane potential to lower membrane potential is calculated by the equation (V_1_-V_2_)/r_j_.

Gap junctions ensure cohesive smooth muscle relaxation in the corpus cavernosum during sexual arousal and facilitating blood engorgement and erection (Carvalho et al., 1993). Research has identified connexin43 (Cx43) as the major protein component of these gap junctions in erectile tissues, including the clitoral corpus cavernosum (Christ, et al., 1993; Pointis, 2006). Disruption to the biophysical mechanisms of gap junctions can significantly impair sexual function, particularly processes like erection (Ma et al., 2024). Varicosities are bead-like enlargements along autonomic nerve fibers that serve as key sites for neurotransmitter release in smooth muscle regulation. Unlike the precise neuromuscular junctions of skeletal muscle, varicosities facilitate a more diffuse mode of transmission, where neurotransmitters are released into the extracellular space and affect a broader area of nearby smooth muscle cells. This diffuse release allows multiple cells within a functional syncytium to be influenced simultaneously. By binding to receptors on smooth muscle cells, the neurotransmitters modulate ion channel activity and intracellular signaling, resulting in changes to the membrane potential—either depolarization or hyperpolarization—which in turn affects the excitability and contractile behavior of the tissue. Varicosities in smooth muscle contain a variety of neurotransmitters and co-transmitters, including acetylcholine, norepinephrine, NO, ATP, and vasoactive intestinal peptide (VIP). As discussed in the previous section, parasympathetic innervation of CSM tissue plays a crucial role in releasing NO, which mediates both smooth muscle contraction and relaxation. Figure 3b shows the distribution of varicosities from the nerve cells in the smooth muscle syncytium, where the individual cells are interconnected via gap junctions. The black dot denotes the varicosities where the neurotransmitter is being released.

Interstitial cells of Cajal (ICCs) are specialized cells that originate from mesenchymal precursors and share a common developmental lineage with smooth muscle cells. Unlike smooth muscle cells, which are primarily geared toward contraction, ICCs possess fewer contractile elements but are distinguished by their abundance of mitochondria, a well-developed endoplasmic reticulum, and unique membrane channels. Morphologically, ICCs feature a spindle-shaped body, a prominent oval nucleus, scant cytoplasm, and branching, dendrite-like processes (Pellegrini et al., 1999). While ICCs are well-known for their role in the GI tract, where they function as pacemaker cells that generate and propagate electrical slow waves to coordinate rhythmic contractions, they are also found in other organs and tissues. These include the bladder, ureteropelvic junction, vas deferens, prostate, penis, mammary gland, uterus, pancreas, and various blood vessels like the portal vein and CSM cells (Lang and Klemm, 2005). There is also evidence to support that ICCs might perform a similar pacemaking function in CSM cells (Fu et al., 2011; Shafik et al., 2006).

## 6 Calcium dynamics

For physiological processes including controlling muscle tone and organ motility, precise control over smooth muscle activity is ensured by this complex balance between Ca^2+^ release and recapture. Excitation-contraction (E-C) coupling, a key concept mentioned in the introduction section, links the changes in membrane potential to alterations in intracellular Ca^2+^ levels, ultimately leading to force generation for contraction in all smooth muscle cells. Variations in intracellular Ca^2+^ concentration act as a molecular switch, triggering downstream signaling pathways that regulate cellular responses to both internal and external stimuli. The extracellular Ca^2+^ input through a variety of ion channels and Ca^2+^ outflow from cytosol through pumps and exchangers are carefully balanced to control the intracellular Ca^2+^ level (Asunción-Alvarez et al., 2024). In addition, the intracellular Ca^2+^ dynamics process relies on two other primary mechanisms for Ca^2+^ release from the sarcoplasmic reticulum (SR): inositol 1,4,5-trisphosphate-induced Ca^2+^ release and Ca^2+^-induced Ca^2+^ release (CICR) (Berridge MJ. 2008). Following the activation of G-protein-coupled receptors, the second messenger inositol 1,4,5-trisphosphate (IP3) binds to its receptor (IP3R) on the SR, triggering the release of Ca^2+^ ions. Simultaneously, CICR occurs when a small influx of Ca^2+^ through L-type Ca^2+^ channels activate ryanodine receptors (RyRs) on the SR, leading to a much larger release of Ca^2+^ in the form of “sparks” and waves (Amberg and Navedo 2013). While both pathways amplify the initial Ca^2+^ signal to regulate muscle contraction, recent studies highlight a more nuanced role for RyRs in certain smooth muscles, where they may also contribute to muscle relaxation by activating Ca^2+^-activated K^+^ channels (Jackson 2021). The regulation of blood flow within the smooth muscle of the clitoral corpus cavernosum is fundamentally governed by Ca^2+^ dynamics, which directly influence smooth muscle tone and vascular engorgement. These finely tuned signaling mechanisms ensure that the clitoral corpus cavernosum can rapidly adjust blood flow during sexual arousal, maintaining the delicate balance between vasoconstriction and vasodilation. Several research groups have illustrated the role of CICR, IP3, and Rynodine-driven Ca^2+^ dynamics in the CSM cells (Levin et al., 1997; Wray and Burdyga., 2010; Lin et al., 2005; Williams and Stephen, 2007).

## 7 CSM membrane potential model

**FIGURE 4:**
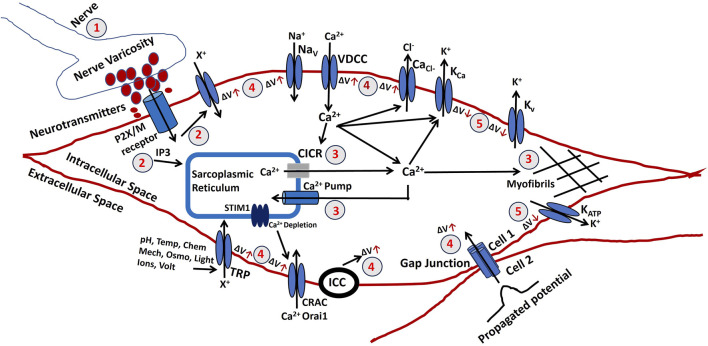
Shows a schematic illustration of the elements that contribute to tension creation in CSM electrophysiology. The numbers correspond to the specific procedures that are described in the text.

Clitoral smooth muscle contraction is fundamentally initiated by intracellular electrical activities, including both slow waves and action potentials, each of which depends on membrane depolarization. As outlined earlier, multiple intrinsic mechanisms—such as ion channel biophysics, gap junction coupling, varicosity-derived neurotransmission, interstitial cells of Cajal activity, and Ca^2+^ handling dynamics—collectively shape the membrane potential. In contrast, subcellular processes that do not directly influence membrane voltage were outside the scope of that discussion. To comprehensively capture the determinants of CSM membrane potential, we have developed a biophysically informed electrophysiological model tailored to clitoral smooth muscle. Figure 4 schematically summarizes the interacting processes that regulate intracellular membrane potential, which is essential for the generation of active contractile tension. These mechanisms, many of which are experimentally validated in other smooth muscle systems, are potentially modulatable and therefore critical for understanding the pathophysiology of abnormal clitoral contractile function. In the figure, red circled numbers indicate sequential stages of membrane potential modulation, with ΔV denoting voltage changes. The red upward arrow represents depolarization (membrane potential rise), whereas the downward arrow indicates hyperpolarization (membrane potential fall).

1. Parasympathetic nerves innervate smooth muscle, releasing neurotransmitters at varicosities. The density and distribution of excitatory nerves, as well as the quantity of neurotransmitter released, affect the membrane potential of the clitoral smooth muscle. These neurotransmitters may include NO, purinergic or cholinergic co-transmitters.
2. An increase in a diffusible second messenger may connect membrane activities with intracellular Ca^2+^ release. In the CSM, purinergic or acetylcholine (ACh) binding to P2X or M3 muscarinic receptors triggers the synthesis of inositol trisphosphate (IP3). It also allows to flow of cation (X^+^) which depolarizes the membrane.
3. Sarcoplasmic Ca^2+^ originates from the SR, an intracellular storage site. Ca^2+^ is transported from the SR to the cytoplasm through channels controlled by intracellular agents. Tension formation depends on factors influencing Ca^2+^ accumulation and release from the SR. Ca^2+^ release from the SR can occur through various mechanisms, including CICR, which is typically initiated by Ca^2+^ flux across the surface membrane. Ca^2+^ is reabsorbed into the SR lumen through an ATP-dependent calcium pump, which transports Ca^2+^ against its concentration gradient. Disruptions in ATP production can impair this process. The final step in generating tension involves raising the Ca^2+^ concentration in the sarcoplasm. Myofibrils require a Ca^2+^ concentration of approximately one mmol/L for half-maximal activation. Ca^2+^ binds to calmodulin, forming a complex that activates myosin through phosphorylation. This interaction between actin and myosin, requiring ATP, initiates contraction.
4. A rise in membrane potential (ΔV) can result from several factors. Membrane potential can propagate between cells via gap junctions, and SWs can be evoked by the pacemaking cells like ICC. Extracellular ATP may bind to P2X receptors, opening non-specific cation channels (X^+^) and increasing membrane potential. This depolarization can activate L-type Ca^2+^ channels (VDCC), leading to Ca^2+^ influx and AP initiation. Voltage-gated Na^+^ channels and Ca^2+^-activated Cl^−^ channels also allow the influx of Na^+^ and efflux of Cl^−^ ions to depolarize the membrane. SOCE is a common mechanism for Ca^2+^ influx activated by a drop in intracellular Ca^2+^ levels in the SR and it also depolarizes the membrane. TRP channels can be activated by various stimuli (pH, temperature, chemicals, mechanical, osmolar, light, ions, and voltage) and allow the influx of cation (X^+^) to depolarize the membrane. Research on CRAC channels led to the identification of Orai1 and STIM1 as primary components, with STIM1 activating both Orai1 and TRPC1. The interaction between TRPC1 and Orai1 is essential for TRPC1 activation.
5. The decline in Ca^2+^ transient following AP or SW generation results in smooth muscle relaxation. The activation of Ca^2+^ channels and generation of APs open various K^+^ channels (K_Ca_, Kvs, K_ATP_) to repolarize the membrane and return it to resting potential. The CSM contraction relaxation cycle completes after the membrane depolarization and AP generation.

## 8 Experimental and computational techniques for studying CSM contraction

Studying the electrophysiology of CSM tissue involves a range of experimental techniques that can provide comprehensive insights into its function and behavior. One approach is the use of patch-clamp electrophysiology, which allows for the precise measurement of ion channel activity and membrane potentials in isolated smooth muscle cells (Hooper and Schmidt, 2017). This technique can also help determine how different pharmacological agents or physiological conditions influence the electrical properties of the cells. In addition to patch-clamp studies, imaging techniques such as fluorescence microscopy combined with voltage-sensitive dyes can offer real-time visualization of electrical activity across larger populations of cells (Müllenbroich, et al., 2021; Shakibi et al., 2025). This method can track the spatial and temporal patterns of electrical signals, providing insights into how smooth muscle cells coordinate their activity. Moreover, genetic and molecular approaches, including the use of knockout or transgenic animal models, can elucidate the roles of specific proteins and signaling pathways in modulating CSM function (Zhong et al., 2024). By integrating these experimental strategies, researchers can build a more complete understanding of the electrophysiological mechanisms governing CSM activity. Immunohistochemistry (IHC) is a valuable tool in studying the electrophysiology of CSM as it allows for the detailed localization and identification of specific proteins within the tissue (Mebratie and Dagnaw, 2024). Contraction measurement complements IHC by offering direct insights into the functional responses of CSM tissue. Techniques such as isometric tension recordings or real-time imaging of muscle contraction can quantify the force and dynamics of muscle contractions in response to various stimuli (Hoppeler, 2014). These measurements are essential for understanding how electrical signals translate into mechanical actions and how different conditions or pharmacological agents affect this process. Our knowledge of excitable cell electrophysiology is also greatly enhanced by computational methods, especially in the fields of calcium kinetics and ion channel biophysics (Mahapatra and Manchanda, 2015). These models allow for the exploration of ion channel kinetics, gating mechanisms, and the impact of mutations or drugs on their function. Computational models can simulate the spatial and temporal distribution of Ca^2+^ in response to electrical stimuli, helping to elucidate the complex interactions between ion channels, receptors, and intracellular signaling pathways. These models can also predict how alterations in Ca^2+^ handling, due to disease or pharmacological intervention, might affect muscle function. By combining computational approaches with experimental data, researchers can develop a comprehensive understanding of the biophysical and physiological processes that underlie clitoral smooth muscle function, leading to more targeted therapeutic strategies. Numerous computational models have been developed to study various aspects of smooth muscle electrophysiology (Mahapatra et al., 2017). Establishing a computational model for CSM electrophysiology could provide valuable insights for identifying new pharmacological targets to address abnormal CSM contractions associated with sexual arousal disorders.

## 9 Pharmacological implications and future directions

Electrophysiological and ion channel therapies have gained significant traction in recent years as targeted treatments for a wide range of physiological disorders. These approaches are especially prominent in managing neurological, cardiac, and muscular channelopathies, where dysfunctions in ion channels disrupt cellular excitability and signaling. Membrane depolarization is essential for the activation of VDCCs and the resulting Ca^2+^ influx, making it a critical target for therapeutic intervention in smooth muscle disorders. For example, in vascular smooth muscle, excessive depolarization contributes to vasospastic conditions such as Raynaud’s phenomenon and cerebral vasospasm, both of which are effectively treated with L-type Ca^2+^ channel blockers (Striessnig and Ortner, 2022). This approach may be applicable to CSM hyperexcitability, where normalizing membrane potential could reduce pathological hypercontractility. While L-type Ca^2+^ channel antagonists like nifedipine have demonstrated efficacy in relaxing genital smooth muscle in preclinical studies, their use has not yet been evaluated in clinical trials for FSAD (Lee et al., 2022). Pharmacological studies on BKCa channels modulators like NS1619, NS11021, NS-8, LDD175, and iberiotoxin have shown the ability to improve penile erectile responses to varying degrees without direct evidence for clitoral tissues (Sancho and Kyle, 2021). Parasympathetic input, delivered via varicosity-mediated release of ACh and NO, plays a pivotal role in regulating CSM tone. Disruption of this neurotransmission has been linked to FSAD, indicating that therapies targeting either presynaptic transmitter release or postsynaptic receptor responsiveness may help restore normal arousal function (Graziottin et al., 2022). At present, most approved pharmacological treatments—including flibanserin, bremelanotide, and testosterone formulations—exert their effects primarily through central neuromodulation rather than direct modulation of peripheral ion channel activity (Belkin et al., 2015). Experimental models, however, have shown that CSM responds to agents such as vasoactive intestinal peptide (VIP), NO donors, and PDE5 inhibitors like tadalafil and sildenafil, providing a mechanistic basis for peripheral therapeutic approaches (Kaltsas et al., 2024).

Pharmacological strategies targeting Ca^2+^ handling pathways represent a compelling approach for treating disorders involving abnormal smooth muscle contractility. For example, agents that enhance ATP production, thereby supporting energy-dependent Ca^2+^ regulation mechanisms like SERCA, or those that directly modulate SR Ca^2+^ channels, may prove especially effective in conditions marked by impaired Ca^2+^ release or uptake. A pertinent clinical example is the use of istaroxime in heart failure. By stimulating SERCA2a activity and reducing its inhibition by phospholamban, istaroxime enhances both Ca^2+^ sequestration and release from the SR, ultimately improving myocardial contractility and relaxation without contributing to arrhythmic risk (Zaza and Marcella, 2025). These translating agents can also enhance SERCA function into urogenital smooth muscle therapy could address disorders marked by sustained CSM tone. When the sensitivity of Ca^2+^ release channels are altered, pharmacological modulation of these messenger pathways may restore normal function. Indeed, pharmacological IP_3_ receptor inhibitors such as Xestospongin C (XeC) have been widely used to attenuate IP_3_-mediated Ca^2+^ release and diminish agonist-induced smooth muscle contraction in experimental settings (Gambardella et al., 2021). SOCE, involving channels such as Orai1 and TRP family members, maintains Ca^2+^ influx when SR stores are depleted. Aberrant SOCE activity contributes to airway hyperresponsiveness in asthma and abnormal vascular reactivity in hypertension (Khalfaoui and Pabelick, 2023). Targeting these channels—already a focus in pulmonary and cardiovascular research—may yield novel treatments for CSM dysfunction where abnormal Ca^2+^ entry sustains pathological contractility. On the other hand, TRP channels, particularly TRPV1 and TRPA1, are emerging targets due to their role in sensory signaling and genital blood flow regulation. However, no TRP channel modulators have reached clinical trials for FSAD to date (Lee et al, 2022).

Despite historical reliance on extrapolated data from postmortem or non-human models, the prospects for experimental studies on human and animal clitoral tissue are increasingly promising. Recent advances in minimally invasive techniques, such as microbiopsy under anesthesia, have enabled *in vivo* ultrastructural analysis of clitoral cavernous tissue in living subjects, revealing age- and disease-related changes in smooth muscle morphology and vascular architecture (Caruso et al., 2011). Additionally, animal models have been successfully employed to study hemodynamic responses, neurotransmitter modulation, and ion channel activity in clitoral and vaginal tissues following pelvic nerve stimulation and pharmacological interventions (Angulo and Hannan, 2022; Marson et al., 2013). With advances in ethical research protocols and imaging modalities, upcoming investigations are poised to combine organ bath assays, primary smooth muscle cell cultures, and optical spectroscopy to refine our understanding of clitoral physiology and identify novel targets for therapeutic intervention. Future efforts may also concentrate on developing advanced diagnostic and therapeutic tools for clitoral smooth muscle disorders. Innovations in imaging techniques, like high-resolution ultrasound and magnetic resonance elastography, could enable non-invasive evaluation of clitoral biomechanics and contractile function. Precision medicine approaches tailored to individual patient needs and disease characteristics hold promise for improving treatment outcomes and minimizing adverse effects (Fountzilas et al., 2022). Collaborative efforts among clinicians, researchers, and industry professionals will be crucial for translating these insights into effective clinical applications, ultimately enhancing the management and treatment of clitoral erection disorders.

## 10 Conclusion

The clitoris, a pivotal structure in female sexual response, relies heavily on the precise regulation of clitoral smooth muscle tone to sustain its functional integrity and responsiveness during arousal. Despite notable advances in the electrophysiology of excitable tissues—particularly in the realms of ion channel biophysics, calcium signaling, and neuroregulation—the electrophysiological study of CSM remains underdeveloped compared to its cardiac and neuronal counterparts. This disparity is largely due to technical limitations in isolating viable clitoral cells and applying high-resolution electrophysiological methods to such tissue. As a result, current understanding of CSM function—especially in regard to calcium kinetics and ion channel modulation—is fragmented. This review integrates experimental, computational, and clinical perspectives to establish a cohesive biophysical framework for CSM dynamics, with implications for diagnosing and treating FSAD. By illuminating the critical role of ion channels, intracellular signaling pathways, and neurotransmitter interactions in modulating muscle tone and contraction, the review identifies actionable targets for pharmacological intervention. Given the historical neglect of female sexual health in clinical research, this focused analysis provides essential insight into the cellular and molecular mechanisms driving clitoral physiology. However, notable gaps remain: subcellular signaling cascades, receptor-ligand interactions, and intracellular feedback mechanisms are only superficially addressed. Further exploration of these domains could yield more precise therapeutic strategies. Additionally, while extrapolation from non-clitoral smooth muscle models—such as vascular or uterine tissues—has served as a proxy for mechanistic interpretation, these analogs exhibit significant variability in receptor distribution, electrophysiological properties, and hormonal sensitivity. Such heterogeneity compromises the translational accuracy of existing models for CSM physiology. Therefore, future research must prioritize direct experimentation with human or validated clitoral tissue to ensure fidelity in therapeutic development and mechanistic understanding. By addressing these limitations, the field can move toward more effective clinical interventions that enhance sexual health and wellbeing in women affected by FSAD.
